# Adjuvant Therapy After Upfront Resection of Resectable Pancreatic Cancer: Patterns of Omission and Use—A Prospective Real-Life Study

**DOI:** 10.1245/s10434-024-14951-4

**Published:** 2024-01-29

**Authors:** Salvatore Paiella, Giuseppe Malleo, Gabriella Lionetto, Alice Cattelani, Fabio Casciani, Erica Secchettin, Matteo De Pastena, Claudio Bassi, Roberto Salvia

**Affiliations:** 1 General and Pancreatic Surgery Unit, Pancreas Institute, University of Verona, Verona, Italy; 2 Department of Surgical Sciences, University of Verona, Verona, Italy

**Keywords:** Pancreatic cancer, Adjuvant therapy, Pancreatic surgery, Failure to rescue, Postoperative complications

## Abstract

**Background:**

Little is known about adjuvant therapy (AT) omission and use outside of randomized trials. We aimed to assess the patterns of AT omission and use in a cohort of upfront resected pancreatic cancer patients in a real-life scenario.

**Methods:**

From January 2019 to July 2022, 317 patients with resected pancreatic cancer and operated upfront were prospectively enrolled in this prospective observational trial according to the previously calculated sample size. The association between perioperative variables and the risk of AT omission and AT delay was analyzed using multivariable logistic regression.

**Results:**

Eighty patients (25.2%) did not receive AT. The main reasons for AT omission were postoperative complications (38.8%), oncologist’s choice (21.2%), baseline comorbidities (20%), patient’s choice (10%), and early recurrence (10%). At the multivariable analysis, the odds of not receiving AT increased significantly for older patients (odds ratio [OR] 1.1, *p* < 0.001), those having an American Society of Anesthesiologists score ≥II (OR 2.03, *p* = 0.015), or developing postoperative pancreatic fistula (OR 2.5, *p* = 0.019). The likelihood of not receiving FOLFIRINOX as AT increased for older patients (OR 1.1, *p* < 0.001), in the presence of early-stage disease (stage I–IIa vs. IIb–III, OR 2.82, *p* =0.031; N0 vs. N+, OR 3, *p* = 0.03), and for patients who experienced postoperative major complications (OR 4.7, *p* = 0.009). A twofold increased likelihood of delay in AT was found in patients experiencing postoperative complications (OR 3.86, *p* = 0.011).

**Conclusions:**

AT is not delivered in about one-quarter of upfront resected pancreatic cancer patients. Age, comorbidities, and postoperative complications are the main drivers of AT omission and mFOLFIRINOX non-use.

ClinicalTrials registration: NCT03788382.

**Supplementary Information:**

The online version contains supplementary material available at 10.1245/s10434-024-14951-4.

Pancreatic cancer (PC) is a world-leading cause of cancer-related death, with an overall 5-year relative survival rate of 12% and an increased annual incidence of 1%.^1^ In the last two decades, robust evidence has demonstrated that adjuvant therapy (AT) leads to improved survival.^2,3^ The 5-year update of the PRODIGE-24 randomized trial recently corroborated the outstanding long-term results of adjuvant mFOLFIRINOX,^4,5^ which has become the standard of care following upfront pancreatectomy since 2019; however, little is known about the applicability of such a therapeutic combination outside controlled trials. Given the high rate of complications associated with pancreatic resections^6^ and the toxicity profile of mFOLFIRINOX, it can be postulated that only patients with outstanding performance status constituted the study population of the PRODIGE-24 trial, thus introducing a significant selection bias in the study results.

Several reports have thus far shown that the AT omission rate ranges from 30 to 50%,^6–8^ and in these cases, the chance of long-term survival is reduced. For example, almost a decade ago, Merkow et al. reported the magnitude and detailed risk factors for AT omission, demonstrating that even non-life-threatening complications (e.g., pneumonia, urinary tract infection, or surgical site infection) may contribute to the AT omission that occurred in 56.4% of patients experiencing a complicated postoperative course.^9^ Notably, that study was published when the most common therapeutic regimen was gemcitabine, which historically showed a significantly better toxicity profile compared with mFOLFIRINOX.^5^ Finally, AT access is considered a quality metric in pancreas surgery to evaluate systems performance at institutional levels.^10^

Identifying preoperative factors associated with AT omission is paramount to maximizing the multimodal treatment of PC and reducing the fraction of patients receiving incomplete therapy (namely, surgery only). In fact, in the current era, some clinicians are more likely to recommend neoadjuvant therapy even for resectable PC (rPC), for patients deemed to be at high risk of failing to be initiated on AT, and to increase patient likelihood of receiving all intended therapy.^11–13^

To the authors’ knowledge, this is the first study to rigorously depict real-life use of AT after PC resection, defining primarily (1) the pattern of AT omission and use; (2) factors associated with AT omission; and (3) factors associated with AT delay. Second, the adherence to national guidelines was assessed.

## Methods

### Study Design

This was a single-center, prospective, observational study conducted from 1 January 2019 through 27 July 2022 at the Unit of Pancreatic Surgery, Pancreas Institute, University of Verona, Verona, Italy. The local Ethics Committee prospectively approved the collection of patient data (PAD-R, #1101CESC). The trial protocol is available at ClinicalTrials.gov (NCT03788382). The authors were responsible for the study design, data analysis, and contents of this article. The principles of the Declaration of Helsinki^14^ and the Strengthening the Reporting of Observational Studies in Epidemiology (STROBE)^15^ guidelines were followed to conduct the trial and report the study, respectively.

### Patient Selection and Data Collection

Preoperative, intraoperative, and postoperative data of consecutive patients who underwent upfront resection (pancreatoduodenectomy, distal pancreatectomy, total pancreatectomy), and a final pathology reporting PC, were collected. Patients who received surgery after neoadjuvant therapy for PC were not included since there is no consensus on administering further chemotherapy after pancreatectomy in this circumstance. Patients who experienced in-hospital death after surgery were also excluded.

Patients received AT at the authors’ institution or in other centers according to the patient’s region of residence and preference. The chemotherapy regimen was assigned at the discretion of the treating oncologist. For patients receiving AT in other institutions, data were obtained by direct contact with the patient or the treating oncologist. In detail, the following information was collected: AT administration (yes/no), reasons for AT omission, time to AT start (days), and type of chemotherapy prescribed.

### Outcomes

The primary endpoints were (1) the proportion of patients not accessing AT at all; (2) the investigation of perioperative factors associated with AT omission; and (3) AT delay. As a secondary endpoint, adherence to the Italian Association of Medical Oncology (AIOM) guidelines was evaluated, considering the introduction in the recommendations of FOLFIRINOX that occurred in October 2019 (9 months after the PRODIGE-24 study). Therefore, for the purposes of this study, from January 2019 to October 2019, gemcitabine was considered the standard of care, while onwards, FOLFIRINOX was considered standard of care.

### Statistical Analysis

A precision-based approach was used to calculate the sample size. Assuming up to 85% of the subjects would have received AT (based on previous historical institutional data), the study would require a sample size of 317 patients to estimate the expected proportion with 5% absolute precision and 95% confidence. Continuous variables were expressed as medians with interquartile range (IQR) and were compared using the non-parametric Mann–Whitney U test. Categorical variables were presented as frequencies with percentages and were compared using the Chi-square test or Fisher’s exact test.

The association between clinicopathological and perioperative data and AT administration was tested using multivariable logistic regression models (backward regression, Wald test, *p* <0.05 for variable entry, *p* > 0.1 for removal). Variables were selected for model entry according to their clinical relevance and statistically significant association with the outcome of interest at univariable analysis (*p* < 0.01). Analysis of the area under the receiving operating characteristic (ROC) curve was performed to identify the best age threshold associated with the likelihood of adjuvant treatment omission, using the Youden Index (J). For AT delay, the cut-off chosen was 12 weeks. Modeling was performed with no missing data.

Statistical analyses were performed using the MedCalc software (MedCalc, Oostende, Belgium)**.**

## Results

**Fig. 1:**
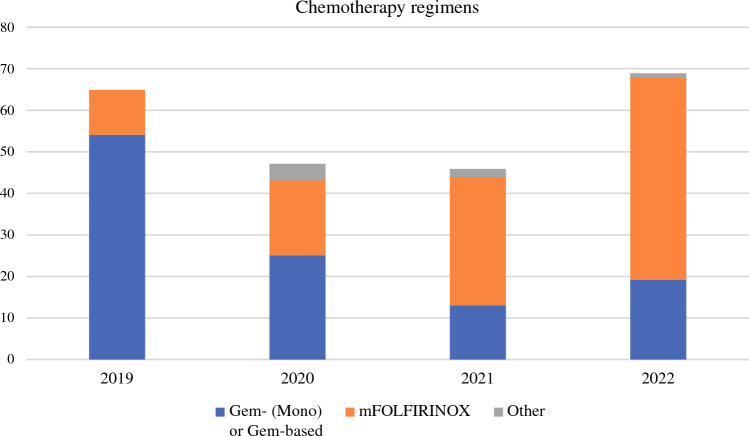
ROC curve analysis for prediction of adjuvant therapy omission (*left*) and mFOLFIRINOX omission (*right*). *ROC* receiver operating characteristic, *AUC* area under the curve

A total of 1122 patients with periampullary disease received pancreatectomy over the study period. After applying the inclusion and exclusion criteria, 317 patients were enrolled as planned. Fig. 1 of Electronic supplementary material (ESM) shows the study flowchart. The median age was 70 years (IQR 9), and the sexes were almost equally balanced. The proportion of ASA class > 2 and Charlson Age Comorbidity Index (CACI) ≥ 4 patients was 31.8% and 30%, respectively. Table 1 reports the general characteristics of the study population.

**Table 1:** General characteristics of the study population [*n* = 317]

Variable	Total
*Demographics*	
Age, years [median (IQR)]	70 (9)
Sex, Female	162 (51.1)
BMI, kg/m^2^ [median, IQR)]	23.7 (4.8)
Underweight—BMI < 18.5	14 (4.5)
Normal—BMI 18.5–25.0	185 (58.3)
Overweight—BMI 25.0–30.0	96 (30.3)
Obese—BMI >30.0	22 (6.9)
Smoking, current or past	179 (56.5)
Alcohol abuse, current or past	45 (14.2)
FH of pancreatic cancer	33 (10.4)
PH of other malignancies	20 (6.3)
ASA score III–IV	101 (31.8)
CACI ≥ 4	95 (30.0)
Rectal colonization by MDR bacteria	27 (8.5)
Biliary stenting	141 (44.5)
Weight loss	192 (60.6)
Anemia	108 (34.1)
Cholangitis within 6 weeks from surgery	20 (6.3)
CA19-9 serum levels, U/mL [median (IQR)]	98 (62)
*Surgical data*	
Pancreaticoduodenectomy	199 (62.8)
Distal splenopancreatectomy	97 (30.6)
Total pancreatectomy	21 (6.6)
Minimally invasive approach	31 (9.8)
Vascular resection	22 (6.9)
Estimated blood loss [median (IQR)]	420 (500)
*Pathology data*	
AJCC staging (8th edition)	
Stage I	42 (13.2)
Stage II	109 (34.4)
Stage III	166 (52.4)
R status, R0	244 (76.8)
Lymph nodes examined [median (IQR)]	39 (21)
N status	
N0	48 (15.1)
N1	104 (32.8)
N2	165 (52.1)

Data are expressed as *n* (%) unless otherwise specified

*CACI* Charlson Age Comorbidity Index^30^, *FH* family history, *PH* personal history, *IQR* interquartile range, *BMI* body mass index, *ASA* American Society of Anesthesiologists, *MDR* multidrug resistance, *AJCC* American Joint Committee on Cancer

### Pattern of Adjuvant Therapy Omission

After surgery, 80/317 patients (25.2%) did not receive AT, whereas 217 patients (74.8%) eventually did receive AT. AT was omitted due to postoperative complications lasting >12 weeks or clinical deterioration leading to an Eastern Cooperative Oncology Group performance status (ECOG PS) score of 2 in 38.8% of cases. Omission due to oncologist’s choice occurred in 21.2% of patients, mainly for early-stage cases (stage I–IIA). Other reasons were baseline comorbidities (CACI >3) in 20% of cases, patient choice (10%), and very early recurrence on postoperative restaging (10%). Table 2 summarizes the oncological data, including reasons for AT omission, time to AT initiation, and the regimens prescribed.

**Table 2:** Oncology data

Variable	Total
Adjuvant therapy omission	80 (25.2)
Pattern of failure	
Postoperative complications	31 (38.8)
Deemed not necessary^a^	17 (21.2)
Baseline comorbidities (CACI ≥4)	16 (20)
Patient’s choice	8 (10)
Early recurrence	8 (10)
Chemotherapy regimen	
Gemcitabine, monotherapy	99 (41.8)
mFOLFIRINOX	91 (38.4)
Gemcitabine-based polychemotherapy	37 (15.6)
Other regimens	10 (4.2)
Time discharge-chemotherapy start, weeks [median (IQR)]	8 (4.0)

Data are expressed as *n* (%) unless otherwise specified

*CACI* Charlson Age Comorbidity Index, *IQR* interquartile range

^a^R0, or Stage I or Stage IIA

### Perioperative Factors Associated with Adjuvant Therapy Omission

Patients not accessing AT were more frequently older, had a higher ASA score (III or IV) and a CACI index, and received a microscopically non-radical (R1) surgery (all *p* < 0.001) [Table 3]. The ROC curve analysis found a cut-off value for not receiving AT of 78 years (AUC 0.686; *p* < 0.001) (Fig. 1).

**Table 3:** Association between perioperative factors and adjuvant therapy access

Variable	Total [*n* = 317]	No adjuvant therapy [*n* = 80]	Adjuvant therapy [*n* = 237]	*p*-value
Baseline data				
Age, years [median (IQR)]	71 (14)	75 (12)	70 (12)	**<0.001**
<60 years	44 (13.9)	3 (3.8)	41 (17.3)	**<0.001**
−69 years	98 (13.9)	18 (22.5)	80 (33.8)	
70–79 years	127 (40.1)	35 (43.8)	92 (38.8)	
≥80 years	48 (15.1)	24 (30)	24 (10.1)	
Sex (male:female)	155:162	44 (55)	111 (46.8)	0.245
BMI, kg/m^2^ [median (IQR)]	23.7 (3.8)	23.6 (4.6)	23.8 (4.8)	0.906
Underweight – BMI <18.5	14 (4.4)	5 (6.3)	9 (3.8)	0.473
Normal – BMI 18.5–25.0	184 (58)	41 (51.9)	143 (60.3)	
Overweight – BMI 25.0–30.0	100 (31.5)	28 (35)	72 (30.4)	
Obese – BMI >30.0	19 (6)	6 (7.5)	13 (5.5)	
Smoking, current or past	179 (56.5)	47 (58.7)	132 (55.3)	0.742
Alcohol abuse, current or past	45 (14.2)	14 (17.5)	31 (13.1)	0.328
FH of pancreatic cancer	33 (10.4)	9 (11.3)	24 (10.1)	0.776
ASA score III–IV	101 (31.9)	38 (47.5)	63 (26.6)	**<0.001**
CACI [median (IQR)]	2 (0)	6 (1)	5 (2)	**<0.001**
Weight loss	192 (60.6)	50 (62.5)	142 (59.9)	0.233
Biliary stenting	141 (44.5)	31 (38.7)	110 (46.4)	0.683
Cholangitis within 6 weeks from surgery	20 (6.3)	4 (5)	16 (6.8	0.233
CA19-9, U/mL [median (IQR)]^a^	98 (162)	115 (230)	97 (152)	0.576
Surgical data				0.417
Pancreatoduodenectomy	199 (62.8)	49 (61.2)	150 (63.3)	0.456
Distal splenopancreatectomy	96 (30.3)	23 (28.7)	73 (30.8)	–
Total pancreatectomy	22 (6.9)	8 (10)	14 (5.9)	–
Minimally invasive approach	31 (9.8)	12 (15)	19 (8)	0.069
Vascular resection	22 (6.9)	6 (7.5)	16 (6.8)	0.820
Estimated blood loss [median (IQR)]	420 (500)	460 (473)	420 (470)	0.683
Pathological data				
Stage I	42 (13.2)	11 (13.8)	31 (13.1)	0.454
Stage II	110 (34.7)	32 (40)	78 (32.9)	–
Stage III	165 (52.1)	37 (46.2)	128 (54)	–
R status, R1	73 (23)	25 (31.2)	48 (20.3)	**0.043**
N status				
N0	48 (15.1)	14 (17.5)	34 (14.3)	0.760
N1	114 (36)	29 (36.2)	85 (35.9)	–
N2	155 (48.9)	37 (46.2)	118 (49.8)	–

Bold values indicate statistically significant

Data are expressed as *n* (%) unless otherwise specified

*CACI* Charlson Age Comorbidity Index^30^, *IQR* interquartile range, *BMI* body mass index, *FH* family history, *PH* personal history, *ASA* American Society of Anesthesiologists, *CACI* Charlson Age Comorbidity Index

^a^*N* = 282 (89.9%); in 35 cases, the CA19-9 was not expressed

Regarding the association with postoperative course, approximately one of two patients experienced at least a postoperative complication (*n* = 149, 50.2%), whereas major complications occurred in 42 patients (13.2%) and cardiopulmonary complications occurred in 55 (17.3%) patients. Patients experiencing a complicated postoperative course were significantly less likely to receive AT (all complications, major complications, clinically relevant pancreatic fistula, abdominal collections requiring treatment, surgical site infections, sepsis and cardiopulmonary events; all *p* < 0.05 (Table 4). In fact, those who did not have access to AT had a longer hospital stay.

**Table 4:** Association between postoperative complications and adjuvant therapy access

Variable	Total [*n* = 317]	No adjuvant therapy [*n* = 80]	Adjuvant therapy [*n* = 237]	*p*-Value
Length of stay [median (IQR)]	10 (11)	13 (28.0)	9 (4.0)	**< 0.001**
Any complication	159 (50.2)	55 (68.7)	104 (43.9)	**< 0.001**
Major complications (CD ≥3)	42 (13.2)	17 (40.5)	25 (10.5)	**0.014**
Clinically-relevant POPF^a^	49 (15.5)	22 (27.5)	27 (1.4)	**< 0.001**
Postpancreatectomy hemorrhage^b^	25 (7.9)	7 (8.8)	18 (7.6)	0.740
Chyle leak^a^	9 (2.8)	3 (3.7)	6 (2.5)	0.571
Delayed gastric emptying^b^	29 (9.1)	8 (10.0)	14 (5.9)	0.213
Re-intervention	24 (7.6)	9 (11.3)	15 (6.3)	0.150
Abdominal collections	40 (12.6)	17 (21.2)	23 (9.7)	**0.007**
Surgical site infection	18 (5.7)	9 (11.3)	9 (3.8)	**0.012**
Sepsis	45 (14.2)	22 (27.5)	23 (9.7)	**< 0.001**
Cardiopulmonary complications^c^	55 (17.4)	23 (28.7)	32 (13.5)	**0.001**

Bold values indicate statistically significant

Data are expressed as *n* (%) unless otherwise specified

*IQR* interquartile range, *CD* Clavien–Dindo^34^, *POPF* postoperative pancreatic fistula, *ISGPF* International Study Group of Pancreatic Fistula, *ISGPS* International Study Group for Pancreatic Surgery

^a^Graded according to the ISGPF^31^

^b^Graded according to the ISGPS^32,33^

^c^Including any cardiopulmonary event requiring treatment

At the multivariable analysis, the likelihood odds of not receiving AT increased significantly for older patients (odds ratio [OR] 1.09, 95% confidence interval [CI] 1.05–1.13, *p* < 0.001), those with an ASA score of III–IV (OR 2.03, 95% CI 1.14–3.6, *p* = 0.015), or developing clinically relevant postoperative pancreatic fistula (OR 2.5, 95% CI 1.15–6.1, *p* = 0.019). Notably, no pathological parameter was associated with AT initiation (Table 5).

**Table 5:** Univariable and multivariable logistic regression for the risk of not receiving adjuvant chemotherapy according to selected variables

Variable	Univariable [OR (95% CI)]	*p*-Value	Multivariable [OR (95% CI)]	*p*-Value
Baseline data				
Age, years	1.09 (1.05–1.12)	**<** **0.001**	1.10 (1.01–1.14)	**<** **0.001**
ASA score				
I–II,	Ref			
III–IV	2.5 (1.48–4.2)	**<** **0.001**	2.03 (1.14–3.6)	**0.015**
CACI	1.51 (1.25–1.82)	**<** **0.001**		
CA19-9, U/mL	4 (1.38–11.53)	**0.010**		
Surgical data	1.00 (0.99–1)	0.437		
Minimally invasive approach				
No	Ref			
Yes	2.02 (0.93–4.38)	0.073		
Pathological data				
Staging				
Stage IA–IIA	Ref			
Stage IIB–III	0.89 (0.44–1.78)	0.749		
R status				
R0	Ref			
R1	1.79 (1.01–3.16)	**0.045**		
N status				
N0	Ref			
N1–2	0.76 (0.38–1.51)	0.437		
Postoperative data				
Any complication, no/yes	2.81 (1.64–4.81)	**<** **0.001**		
Major complication (CD ≥3), no/yes	2.28 (1.16–4.5)	**0.016**		
Abdominal collection, no/yes	2.51 (1.26–4.99)	**0.008**		
Surgical site infection, no/yes	3.21 (1.22–8.4)	**0.017**		
Sepsis, no/yes	3.52 (1.93–6.77)	**<** **0.001**		
Cardiopulmonary complication, no/yes	2.58 (1.4–4.76)	**0.002**		
CR-POPF	2.95 (1.56–5.55)	**<** **0.001**	2.5 (1.15–6.18)	**0.019**

Bold values indicate statistically significant

*OR* odds ratio, *CI* confidence interval, *CACI* Charlson Age Comorbidity Index^30^, *CD* Clavien–Dindo^34^, *CR-POPF* clinically relevant postoperative pancreatic fistula, *ASA* American Society of Anesthesiologists

### Guidelines Adherence

**Fig. 2:**
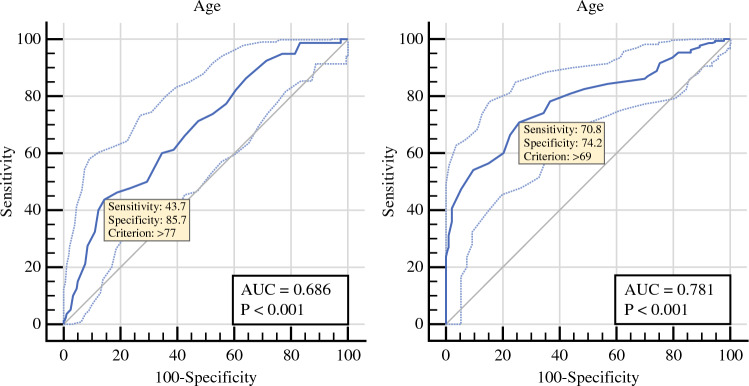
Chemotherapy regimens prescribed during the study period (for guideline adherence evaluation; study period November 2019–July 2022). *Gem* gemcitabine, *mono* monotherapy

Adjuvant FOLFIRINOX utilization increased approximately fivefold over the study period (from 16.1% to 71%) (Fig. 2). Patients not receiving FOLFIRINOX were significantly older (median age 65 vs. 74 years, *p* < 0.001), had a history of alcohol abuse, higher ASA and CACI score, and displayed more advanced N stage (all *p* < 0.05) [electronic supplementary material (ESM) Table S1]. The ROC curve analysis found the cut-off value for not receiving AT with mFOLFIRINOX was > 69 years (AUC 0.781, *p* < 0.001) (Fig. 1).

At the multivariable analysis, the likelihood of not receiving mFOLFIRINOX increased for older patients (OR 1.10, 95% CI 1.06–1.14, *p* < 0.001), patients who experienced major complications during the postoperative course (OR 4.7, 95% CI 1.46–15.3, *p* = 0.009), and in less advanced cases, either in terms of stage (stage I–IIa vs. stage IIb–III: OR 2.82 95% CI 1.09–7.25, *p* =0.031) or nodal involvement (N0 vs. N+, OR 3, 95% CI 1.11–8.53, *p* = 0.030) [ESM Table S2].

### Adjuvant Therapy Delay

Overall, 237 patients initiated AT after a median of 8 weeks from surgery (IQR 6–10), and 22 (9.3%) eventually did initiate AT after 12 weeks. On multivariable analysis, major complications were associated with a more than twofold increased likelihood of delay in AT (OR 3.86, 95% CI 1.35–11.05, *p* = 0.011) [ESM Table S3].

## Discussion

The main aim of this study was to depict a real-life scenario of the use of AT after upfront PC resection, focusing on its omission and the associated factors. A prospective data collection was performed to investigate this aspect thoroughly. In line with some single-center findings,^10,16–19^ we found that one-quarter of patients (25.2%) do not receive any chemotherapy after surgery—a significantly lower rate when compared with previous registry- or population-based reports where this proportion settles at around 40–50%.^9,20–22^

We report that postoperative complications predicted AT therapy and, specifically, FOLFIRINOX omission. Clinical deterioration following a complicated postoperative course has been found to have a pivotal role in negatively influencing access to AT.^9,22–24^ Of note, in a recent multicenter study, Henry et al. demonstrated that the detrimental effect of major postoperative complications on survival is largely mediated by AT omission.^25^ The strict association between a complicated postoperative course and the likelihood of AT omission also impacts treatment selection. Indeed, the patient allocation to either a treatment or another (in the case of resected PC, upfront surgery vs. neoadjuvant therapy) also needs to be weighed on the likelihood of recovering from possible major complications. In this sense, large-scale studies focusing on the identification of patients at higher risk of failure to rescue after pancreatic surgery,^26^ effective prehabilitation strategies, or a bundle of perioperative interventions to improve the recovery,^27^ are eagerly awaited.

Failure to recover from complications and baseline comorbidities accounted for about 60% of AT omission causes. Age might be the trade union between these two factors as it has traditionally been found to be a strong driver of AT omission.^16,18,20,22,28^ In the present study, increasing age remained independently associated with AT omission or not receiving FOLFIRINOX (*p* < 0.001). The likelihood of not receiving AT steadily increased with age, reaching up to 50% in octogenarians. A previous study focusing on AT use in octogenarians reported similar findings (47.4% of AT use), yet it demonstrated the beneficial oncological effects on survival.^29^ Of note, while the ROC curve analysis found a cut-off of 78 years for not receiving AT (AUC 0.686, *p* < 0.001), that cut-off decreased to 70 years for not receiving FOLFIRINOX (AUC 0.781, *p* < 0.001). This relatively low cut-off, combined with the finding that having an ASA score of III–IV led to a more than twofold increased likelihood of not receiving FOLFIRINOX, made us speculate that in the presence of a septuagenarian patient with relevant comorbidities, at the time of preoperative consultation, a multidisciplinary evaluation with a geriatric and oncologic assessment is desirable. More in general, a comprehensive baseline evaluation integrating surgical, anesthesiologic, oncologic, psychologic and nutritional assessment could help in identifying patients at high risk of futile operations and selecting the most appropriate treatment algorithm, including the option of neoadjuvant therapy for those who are expected not to receive AT.^31,32^

Another surprising finding of this study is that in 21.2% of cases, the oncologist decided not to prescribe any AT due to early-stage PC. This result, already reported by Xia et al.,^18^ may seem inexplicable given both the tumor biology of PC, which is thought to be aggressive and micrometastatic since the very early stages, and current clinical guideline recommendations. However, given the rarity of such a condition, data on the efficacy of AT after resection of early-stage PC are scant,^33^ and no definitive conclusion could be derived about its actual benefit.

Regarding the compliance to guidelines rate, our findings report that FOLFIRINOX prescription increased fivefold during the study period (from 16 to 71%), reflecting a steadily increased acceptance of the guideline recommendation. This is concordant with the recently published GARIBALDI survey, promoted by the AIOM, which presented an overall guidelines adherence of 69% in resected PC patients. However, both the survey and this study were developed at the point of introducing FOLFIRINOX as first-option AT into practice, which occurred in October 2019, compared with the results of PRODIGE-24 published in January 2019.^34^ Incorporating guidelines require time to complete as clinical experience and caseload increase. Thus, any further consideration may become inappropriate.

This study has some limitations. First, given the overall number of resected PCs undergoing neoadjuvant therapy, a selection bias applies to the cohort of patients who were selected for surgery; however, the authors could not critically find major drivers of patient selection. Second, the oncological consultation was not centralized at the authors’ institution. Third, we could not collect perioperative nutritional, performance status, and laboratory data, which may have driven the oncological decision not to prescribe AT. Finally, we do not present data on AT completion, tolerance and dose modification, as well as survival data to support the importance of AT completion. Such endpoints will be evaluated in a separate study after concluding appropriate patient follow-up.

## Conclusion

One-quarter of patients who underwent upfront pancreatectomy for resected PC still do not receive AT due to a mix of age- and comorbidity-dependent factors, but primarily due to an adverse postoperative course. There was a steady uptake of adjuvant FOLFIRINOX over the study period. The implications of dose density/intensity and chemotherapy completion on oncologic outcomes warrant future investigation.

### Supplementary Information

Below is the link to the electronic supplementary material.

Supplementary file 1

## Data Availability

The data supporting this study’s findings are available from the corresponding author (SP) upon reasonable request.
