# Supplementary material for: Adjuvant Therapy After Upfront Resection of Resectable Pancreatic Cancer: Patterns of Omission and Use—A Prospective Real-Life Study
Source: Ann Surg Oncol. 2024 Jan 29;31(5):2892–901. doi: 10.1245/s10434-024-14951-4 (PMC10997715; doi:10.1245/s10434-024-14951-4)
Supplement: Supplementary file 1 [file 10434_2024_14951_MOESM1_ESM.docx]

**Supplementary content**

*Perioperative data collected and surgical procedures*

Preoperative data included age, gender, body mass index (BMI), smoking and alcohol habits, Ca 19.9 levels, the American Society of Anesthesiologists physical status classification system (ASA), the Charlson Age Comorbidity Index^1^, and three systemic inflammation indexes: the Neutrophil to Lymphocyte Ratio (NLR), the Platelet to Lymphocyte Ratio (PLR) e the Systemic Immune-Inflammation Index (SII)^2^. Additional information collected for cephalic PCs were: the presence and the type (endoscopically-placed plastic or metallic stent, or the presence of a Percutaneous transhepatic biliary drainage (PTBD) of biliary stents, and any previous cholangitis occurring 6 weeks before surgery. All patients received a preoperative rectal swab for multi-drug resistant bacteria detection and tailored perioperative antibiotic prophylaxis, according to Institutional pathways^3^ and ongoing trials (#NCT04199494).

Intraoperative data collected were: type of surgery (pancreaticoduodenectomy, distal pancreatectomy with splenectomy, or total pancreatectomy), vascular resection (if any), estimated blood loss (EBL), and intraoperative transfusion. For cephalic PCs, data on the Fistula Risk Score (FRS)^4, 5^, routinely adopted to manage drain placement and removal, and to decide the type of pancreatic anastomosis^6^, was collected.

Postoperatively, the following data were collected: length of stay (days), any complication (surgical and non-surgical), major complications (Clavien-Dindo^7^ >2), reintervention, ISGPS- and ISGPF-defined and graded complications such as pancreatic fistula^8^, chile leak^9^, delayed gastric emptying^10^, and post-pancreatectomy hemorrhage^11^, surgical site infections^12^, biliary fistula, duodenojejunal/gastrojejunal fistula, cardio-pulmonary complications, and sepsis.

Pathology data included: R-status (defined based on the presence of tumor cells within 1mm from any resection margin), T-status, N-status, number of lymph nodes harvested, and number of metastatic lymph nodes. The 8th edition of the AJCC staging system was consistently applied.

**Supplementary Figure**

**Figure S1.** Study’s flow diagram.

**
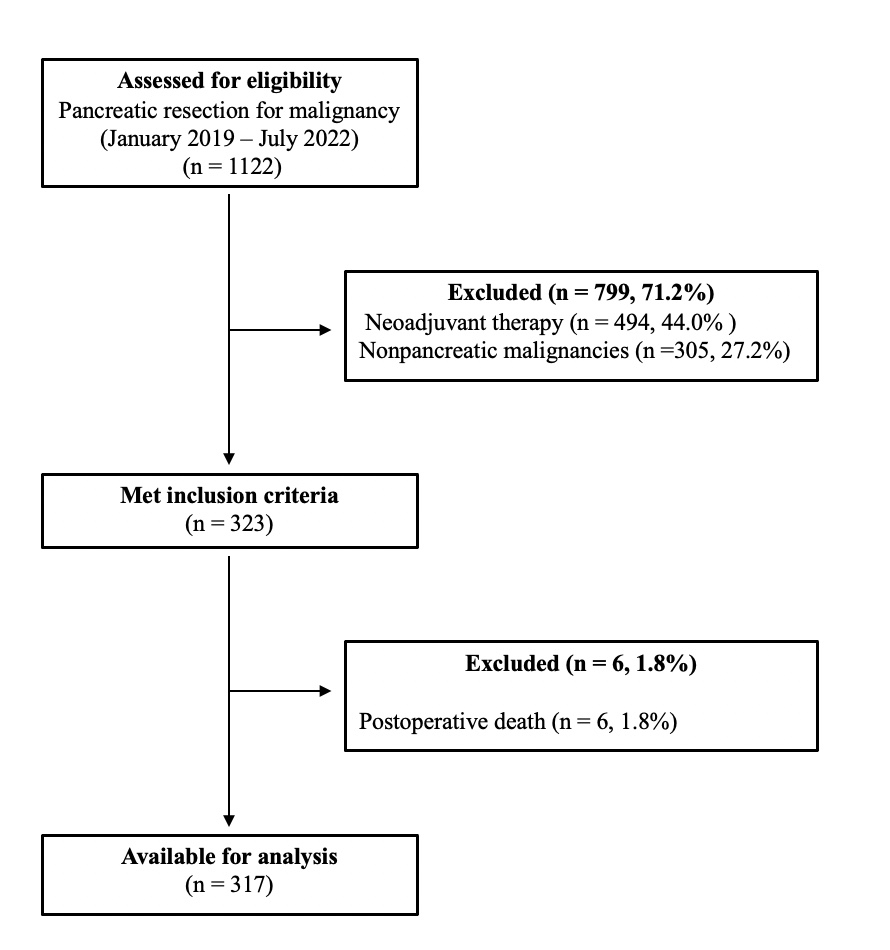
**

**Supplementary Tables**

**Table S1. Association between perioperative factors and mFOLFIRINOX use***

| **Variable** | **mFOLFIRINOX,**  **n=89*** | **Other regimens,**  **n=198** | **p-value** |
| --- | --- | --- | --- |
| *Baseline data*  Age, years (median, IQR)  <60 years, n (%)  60-69 years, n (%)  70-79 years, n (%)  ≥80 years, n (%)  Gender (M:F) | 65 (11)  22 (24.7)  44 (49.4)  23 (25.8)  0  44:45 | 74 (9)  17 (8.6)  43 (21.7)  92 (46.5)  46 (23.2)  97:101 | **<0.001**  **<0.001**  0.366 |
| BMI (kg/m^2^), median (IQR) | 23.6 (4) | 23.9 (4.6) | 0.470 |
| Underweight (BMI <18.5), n (%) | 4 (4.5) | 9 (4.6) | 0.469 |
| Normal (BMI 18.5-25.0), n (%) | 54 (60.7) | 113 (57.4) |  |
| Overweight (BMI 25.0-30.0), n (%) | 27 (30.3) | 60 (30.5) |  |
| Obese (BMI >30.0), n (%) | 4 (4.5) | 15 (7.6) |  |
| Smoking (current or past), n (%)  Alcohol abuse (current or past), n (%)  ASA score III-IV, n (%)  CACI, median (IQR) (%)  Weight loss, n (%)  Biliary stenting, n (%)  Cholangitis within 6 w from surgery, n (%)  Ca 199, U/mL (median, IQR)^**^  *Surgical data*  Pancreatoduodenectomy, n (%)  Distal splenopancreatectomy, n (%)  Total pancreatectomy, n (%)  Minimally invasive approach, n (%)  Vascular resection, n (%)  Estimated blood loss, median (IQR)  *Pathological data*  Stage I, n (%)  Stage II, n (%)  Stage III, n (%)  R-status, R1, (n, %)  N-status  N0 (n, %)  N1 (n, %)  N2 (n, %) | 51 (57.3)  6 (6.7)  19 (21.3)  4 (2)  56 (62.9)  41 (46.1)  6 (6.7)  81 (177)  56 (62.9)  30 (33.7)  3 (3.4)  13 (14.6)  9 (10.1)  450 (562)  6 (6.7)  30 (33.7)  53 (59.6)  20 (22.5)  6 (6.7)  35 (39.3)  48 (53.9) | 109 (55.1)  36 (18.2)  74 (37.4)  5 (2)  114 (57.6)  86 (43.4)  12 (6.1)  105 (172)  126 (63.6)  56 (28.3)  16 (8.1)  18 (9.1)  13 (6.6)  435 (415)  31 (15.7)  69 (34.8)  98 (49.5)  50 (25.3)  37 (18.7)  68 (34.3)  93 (47) | 0.722  **0.011**  **0.007**  **<0.001**  0.394  0.678  0.826  0.571  0.417  -  -  -  0.164  0.297  0.726  0.082^§^  -  -  -  0.612  **0.032**  -  -  - |

^* For this analysis, the study period considered started from the incorporation of mFOLFIRINOX in the Guidelines at the end^

^of October 2019^

^** n=253, 88.1% (in 34 cases, the Ca 199 was not expressed)^

CACI: Charlson Age Comorbidity Index^1^

^§^ Cochran-Armitage test p=0.035

**Table S2. Uni- and multivariable logistic regression for the risk of not receiving**

**FOLFIRINOX according to selected variables**

| **Variable** | **Univariable**  **OR (95% CI)** | **p-value** | **Multivariable**  **OR (95% CI)** | **p-value** |
| --- | --- | --- | --- | --- |
| *Baseline data*  Age, years | 1.10 (1.06-1.14) | **<0.001** | 1.10 (1.06-1.14) | **<0.001** |
| ASA score  I-II,  III-IV  CACI  Ca 199, U/mL  *Surgical data*  Minimally invasive approach  No  Yes  *Pathological data*  Staging,  Stage IA-IIA  Stage IIB-III  R-status,  R0  R1  N-status  N0  N1-2  *Postoperative data*  Any complication, no/yes  Major complication (CD ≥3), no/yes  Abdominal collection, no/yes  Surgical site infection, no/yes  Sepsis, no/yes  Cardiopulmonary complication, no/yes  CR-POPF^#^ | Ref  1.59 (0.86-2.92)  1.85 (1.47-2.33)  0.99 (0.99-1)  Ref.  0.27 (0.09-0.74)  Ref.  0.33 (0.13-0.79)  Ref.  0.80 (0.42-1.53)  Ref.  0.36 (0.15-0.87)  1.7 (1.02-2.82)  2.89 (1.04-8)  1.25 (0.51-3.1)  -^*^  1.25 (0.51-3.1)  3.26 (1.28-8.25)  0.8 (0.35-1.8) | 0.135  **<0.001**  0.707  **0.011**  **0.013**  0.517  **0.023**  0.547  **0.040**  0.615  -  0.615  **0.012**  0.590 | 2.82 (1.09-7.25)  3 (1.11-8.53)  4.7 (1.46-15.3) | **0.031**  **0.030**  **0.009** |

CACI: Charlson Age Comorbidity Index^1^; CD: Clavien-Dindo^7^; ^#^ CR-POPF: clinically-relevant postoperative pancreatic fistula

^*: not computable^

**Table S3. Uni- and multivariable logistic regression for the risk of adjuvant therapy delay (>12 weeks from surgery) according to selected variables**

| **Variable** | **Univariable**  **OR (95% CI)** | **p-value** | **Multivariable**  **OR (95% CI)** | **p-value** |
| --- | --- | --- | --- | --- |
| *Baseline data*  Age, years | 1 (0.95-1.04) | 0.985 |  |  |
| ASA score  I-II,  III-IV  CACI  *Surgical data*  Minimally invasive approach  No  Yes  *Postoperative data*  Any complication, no/yes  Major complication (CD ≥3), no/yes  Abdominal collection, no/yes  Surgical site infection, no/yes  Sepsis, no/yes  Cardiopulmonary complication, no/yes  CR-POPF^#^ | Ref  1.32 (0.51-3.41)  1.27 (0.93-1.72)  Ref.  0.52 (0.06-4.1)  2.4 (0.97-6.03)  3.86 (1.35-11)  2.29 (0.7-7.47)  2.97 (0.57-15.2)  3.21 (1.06-9.74)  1.01 (0.28-3.63)  2.58 (0.86-7.67) | 0.560  0.122  0.535  0.058  **0.011**  0.156  0.192  **0.038**  0.984  0.088 | 3.86 (1.35-11.05) | **0.011** |

CACI: Charlson Age Comorbidity Index^1^; CD: Clavien-Dindo^7^; ^#^ CR-POPF: clinically-relevant postoperative pancreatic fistula

**Supplementary Material References**

1. Charlson ME, Pompei P, Ales KL, et al. A new method of classifying prognostic comorbidity in longitudinal studies: development and validation. *J Chronic Dis* 1987; 40(5):373-83.

2. Hu B, Yang XR, Xu Y, et al. Systemic immune-inflammation index predicts prognosis of patients after curative resection for hepatocellular carcinoma. *Clin Cancer Res* 2014; 20(23):6212-22.

3. De Pastena M, Paiella S, Azzini AM, et al. Preoperative surveillance rectal swab is associated with an increased risk of infectious complications in pancreaticoduodenectomy and directs antimicrobial prophylaxis: an antibiotic stewardship strategy? *HPB (Oxford)* 2018; 20(6):555-562.

4. Callery MP, Pratt WB, Kent TS, et al. A prospectively validated clinical risk score accurately predicts pancreatic fistula after pancreatoduodenectomy. *J Am Coll Surg* 2013; 216(1):1-14.

5. Miller BC, Christein JD, Behrman SW, et al. A multi-institutional external validation of the fistula risk score for pancreatoduodenectomy. *J Gastrointest Surg* 2014; 18(1):172-79; discussion 179-80.

6. Andrianello S, Marchegiani G, Malleo G, et al. Pancreaticojejunostomy With Externalized Stent vs Pancreaticogastrostomy With Externalized Stent for Patients With High-Risk Pancreatic Anastomosis: A Single-Center, Phase 3, Randomized Clinical Trial. *JAMA Surg* 2020; 155(4):313-321.

7. Dindo D, Demartines N, Clavien PA. Classification of surgical complications: a new proposal with evaluation in a cohort of 6336 patients and results of a survey. *Ann Surg* 2004; 240(2):205-13.

8. Bassi C, Marchegiani G, Dervenis C, et al. The 2016 update of the International Study Group (ISGPS) definition and grading of postoperative pancreatic fistula: 11 Years After. *Surgery* 2017; 161(3):584-591.

9. Besselink MG, van Rijssen LB, Bassi C, et al. Definition and classification of chyle leak after pancreatic operation: A consensus statement by the International Study Group on Pancreatic Surgery. *Surgery* 2017; 161(2):365-372.

10. Wente MN, Bassi C, Dervenis C, et al. Delayed gastric emptying (DGE) after pancreatic surgery: a suggested definition by the International Study Group of Pancreatic Surgery (ISGPS). *Surgery* 2007; 142(5):761-8.

11. Wente MN, Veit JA, Bassi C, et al. Postpancreatectomy hemorrhage (PPH): an International Study Group of Pancreatic Surgery (ISGPS) definition. *Surgery* 2007; 142(1):20-5.

12. Horan TC, Gaynes RP, Martone WJ, et al. CDC definitions of nosocomial surgical site infections, 1992: a modification of CDC definitions of surgical wound infections. *Infect Control Hosp Epidemiol* 1992; 13(10):606-8.
